# Persistent Inflammation, Maladaptive Remodeling, and Fibrosis in the Kidney Following Long COVID-like MHV-1 Mouse Model

**DOI:** 10.3390/diseases13080246

**Published:** 2025-08-05

**Authors:** Rajalakshmi Ramamoorthy, Anna Rosa Speciale, Emily M. West, Hussain Hussain, Nila Elumalai, Klaus Erich Schmitz Abe, Madesh Chinnathevar Ramesh, Pankaj B. Agrawal, Arumugam R. Jayakumar, Michael J. Paidas

**Affiliations:** 1 Department of Obstetrics, Gynecology and Reproductive Sciences, University of Miami Miller School of Medicine, Miami, FL 33136, USA; rxr1310@med.miami.edu (R.R.); annarosa.speciale@unifi.it (A.R.S.); emw123@miami.edu (E.M.W.); nilanandhie@gmail.com (N.E.); 2 Department of Health Sciences, Obstetrics and Gynecology Branch, University of Florence, Largo Brambilla 3, 50134 Florence, Italy; 3 Department of Internal Medicine^,^ HCA Florida Kendall Hospital, Miami, FL 33175, USA; hussainhussainmd77@gmail.com; 4 Division of Neonatology, Department of Pediatrics, Holtz Children’s Hospital, University of Miami Miller School of Medicine, Jackson Health System, Miami, FL 33136, USA; kes295@med.miami.edu (K.E.S.A.); mcr314@med.miami.edu (M.C.R.); pagrawal@miami.edu (P.B.A.); 5 Department of Medical Education, University of Miami Miller School of Medicine, Miami, FL 33136, USA

**Keywords:** MHV-1 infection, COVID, inflammation, kidney damage, mRNA sequencing, differential gene expression, fibrosis

## Abstract

Background: Accumulating evidence indicates that SARS-CoV-2 infection results in long-term multiorgan complications, with the kidney being a primary target. This study aimed to characterize the long-term transcriptomic changes in the kidney following coronavirus infection using a murine model of MHV-1-induced SARS-like illness and to evaluate the therapeutic efficacy of SPIKENET (SPK). Methods: A/J mice were infected with MHV-1. Renal tissues were collected and subjected to immunofluorescence analysis and Next Generation RNA Sequencing to identify differentially expressed genes associated with acute and chronic infection. Bioinformatic analyses, including PCA, volcano plots, and GO/KEGG pathway enrichment, were performed. A separate cohort received SPK treatment, and comparative transcriptomic profiling was conducted. Gene expression profile was further confirmed using real-time PCR. Results: Acute infection showed the upregulation of genes involved in inflammation and fibrosis. Long-term MHV-1 infection led to the sustained upregulation of genes involved in muscle regeneration, cytoskeletal remodeling, and fibrotic responses. Notably, both expression and variability of *SLC22* and *SLC22A8*, key proximal tubule transporters, were reduced, suggesting a loss of segment-specific identity. Further, *SLC12A1*, a critical regulator of sodium reabsorption and blood pressure, was downregulated and is associated with the onset of polyuria and hydronephrosis. SLC transporters exhibited expression patterns consistent with tubular dysfunction and inflammation. These findings suggest aberrant activation of myogenic pathways and structural proteins in renal tissues, consistent with a pro-fibrotic phenotype. In contrast, SPK treatment reversed the expression of most genes, thereby restoring the gene profiles to those observed in control mice. Conclusions: MHV-1-induced long COVID is associated with persistent transcriptional reprogramming in the kidney, indicative of chronic inflammation, cytoskeletal dysregulation, and fibrogenesis. SPK demonstrates robust therapeutic potential by normalizing these molecular signatures and preventing long-term renal damage. These findings underscore the relevance of the MHV-1 model and support further investigation of SPK as a candidate therapy for COVID-19-associated renal sequelae.

## 1. Introduction

Multi-organ injury is a hallmark of severe COVID-19, commonly affecting the renal, cardiovascular, pulmonary, and neuropsychiatric systems. Longitudinal studies indicate that 80–85% of patients experience persistent symptoms consistent with post-acute sequelae of SARS-CoV-2 infection even 12 months after the initial illness [1]. Among these complications, acute kidney injury (AKI) has emerged as a frequent and clinically significant outcome in hospitalized COVID-19 patients. The kidneys are particularly susceptible to SARS-CoV-2 due to high expression of ACE2 receptors in proximal tubular epithelial cells, parietal epithelial cells, mesangial cells, and podocytes [2]. In addition to direct viral tropism, indirect mechanisms such as cytokine, immune dysregulation, and underlying comorbidities further increase the risk of AKI.

Renal dysfunction has been observed in approximately 30% of patients following acute and long-term post-SARS-CoV-2 infection. Maladaptive repair following AKI may result in interstitial fibrosis and tubular atrophy, predisposing patients to chronic kidney disease (CKD) and, in severe cases, progression to end-stage renal disease (ESRD). A 2020 meta-analysis further highlighted that severe COVID-19 increases the long-term risk of CKD due to persistent tubular injury, vascular damage, or glomerular diseases such as podocytopathy and collapsing glomerulopathy [3]. Despite these findings, no targeted therapy currently exists for COVID-19-associated AKI, and some patients require renal replacement therapy [4]. Understanding the underlying mechanisms of SARS-CoV-2-induced kidney injury remains essential for identifying therapeutic targets. Also, the exact pathophysiology of renal injury following SARS-CoV-2 infection remains unclear. Human studies have reported the presence of viral particles within the renal glomeruli post-COVID-19, suggesting their potential involvement in triggering pathological changes.

We previously reported that infecting A/J mice with murine hepatitis virus-1 (MHV-1) causes a SARS-like illness with high mortality and multisystem involvement, closely resembling SARS-CoV-2-induced pathology in humans, aside from differences in viral receptor binding [5,6,7,8,9,10]. The MHV-1 model has proven to be a relevant and effective system for studying COVID-19-related organ injury, especially renal complications [11]. Our earlier studies, we found that MHV-1 infection led to acute and chronic kidney injury, including acute tubular injury, collapsing glomerulopathy, proximal and distal tubular necrosis, vascular congestion, epithelial degeneration, vacuolation, interstitial hemorrhage, and inflammation characterized by macrophage and lymphocyte infiltration [7,8,9,10]. Long-term infection was associated with severe tubular necrosis and interstitial edema [7,10]. Additionally, we previously reported increased expression of inflammatory and fibrotic markers in long-term MHV-1 infection at both the mRNA and protein levels, clearly indicating fibrosis in the kidney, which was prevented by SPK treatment [8,10,11,12]. This study aimed to investigate molecular-level changes in the kidneys of acute and long-term MHV-1-infected mice using RNA sequencing. We also explored the effect of SPK, a small synthetic peptide, in preventing kidney damage at the molecular level.

## 2. Materials and Methods

### 2.1. Animal Model and Housing Conditions

Eight-week-old female A/J mice (22–24 g) were obtained from Jackson Laboratory (Bar Harbor, ME, USA) and housed under pathogen-free conditions at the University of Miami Miller School of Medicine animal facility. Mice were maintained on standard irradiated laboratory chow (Envigo 2918, Teklad Diet, Dublin, VA, USA) with autoclaved tap water provided *ad libitum*. All experimental procedures were approved by the Institutional Animal Care and Use Committee (IACUC Protocol #20-131 LF).

### 2.2. MHV-1 Infection and SPIKENET Administration

Murine Hepatitis Virus-1 (MHV-1; ATCC VR-261, Manassas, VA, USA) induced a viral infection. The mice were divided into two groups for acute MHV-1 infection: (1) healthy control and (2) MHV-1-infected. Similarly, the mice were randomized into three groups for long-term MHV-1 infection: (1) healthy controls, (2) MHV-1-infected, and (3) MHV-1-infected with SPK treatment. SPK is our newly designed synthetic peptide, consisting of 15 amino acids (VRIKPGTANKPSED), that specifically binds to the viral S1 glycoprotein (MHV-1 and SARS-CoV-2) and prevents the virus from entering host cells [8,12]. As previously described [5,6,7,8,9,10,11,12,13,14], mice in groups 2 and 3 were intranasally inoculated with 5000 PFU of MHV-1 under light anesthesia. Mice in the third group received 5 mg/kg of SPK intraperitoneally, following protocols established in earlier studies [5,6,7,8,9,10,11,12,13,14]. At designated endpoints—7 days post-infection for acute studies and 12 months post-infection for long-term studies—kidneys were harvested. One half of each kidney was fixed in 10% formalin, and the other half was stored at −80 °C for molecular analysis.

### 2.3. Immunofluorescence

Formalin-fixed paraffin-embedded kidney tissue sections from healthy controls, acute and long-term MHV-1 infected, and MHV-1 infected and SPK-treated samples were incubated with primary antibodies to S1 (cat# FTX635671, monoclonal antibody, GeneTex, Irvine, CA, USA); NC (cat# MA5-29981, monoclonal antibody, Invitrogen, Waltham, MA, USA); IL-6 (cat# SC-1265-R, polyclonal antibody, Santa Cruz, TX, USA); IL-18 (cat# 60070-1, monoclonal antibody, Proteintech, IL, USA); IL-1β (cat# SC-1251, polyclonal antibody, Santa Cruz, TX, USA); and TNF-α (cat# SC1349, polyclonal antibody, Santa Cruz, TX, USA) at a dilution of 1:200. The sections were then incubated with respective secondary antibodies (Alexa Fluor 488 or Alexa Fluor 546 anti-mouse, rabbit, or goat IgG, Life Technologies, San Diego, CA, USA) at dilutions of 1:200 to 1:300. Fluorescent images were randomly captured using a Leica Thunder imager (Leica Microsystems Inc., Deerfield, IL, USA) and analyzed with Velocity 6.0 High-Performance Cellular Imaging Software (PerkinElmer, Waltham, MA, USA), as described previously. Data were normalized to the number of DAPI-positive cells, as well as to the area and intensity of DAPI.

### 2.4. RNA Isolation and Quality Assessment

Total RNA was extracted from kidney tissues using the RNeasy Micro Kit (cat# 74004, Qiagen, MD, USA) and quantified using a NanoDrop OneC spectrophotometer (Thermo Fisher Scientific, Waltham, MA, USA). RNA integrity was evaluated using Qubit high-sensitivity RNA integrity and quality (IQ) Assay Kit (Cat# Q33221, Thermo Fisher Scientific, Waltham, MA, USA).

### 2.5. Library Preparation and Sequencing

The mRNA libraries were prepared using the Illumina Stranded mRNA Preparation Ligation Kit (Cat# 20040534, Illumina Inc., San Diego, CA, USA) according to the protocol recommendations. Library quantification was performed using the Qubit dsDNA BR Assay Kit (Cat# Q32850, Thermo Fisher Scientific, Waltham, MA, USA). Each DNA library was converted to a micromolar concentration and then adjusted to a 10 nM/10 mL solution. The libraries were pooled, denatured, and diluted to 1.3 pM with a 5% PhiX spike-in, then sequenced on the Illumina MiniSeq platform using a 150 bp paired-end high-output kit (Cat# 20000420, Illumina Inc., San Diego, CA, USA).

### 2.6. RNA Sequence Analysis

Gene-level read counts were quantified using feature counts and then annotated using the latest Ensembl mouse annotation (GRCm38.R113). Raw data were trimmed with Trimmomatic (version 0.39, default parameters), a tool for Illumina NGS data. To identify differentially expressed genes, we used three algorithms: DESeq2 (version 1.26.0), edgeR (version 3.28.1), and Limma (version 3.42.2) Bioconductor packages with default settings. Count tables were normalized to TPM (transcripts per million) for visualization and quality control. Sample clustering, pathway analyses, and integration of results were performed using a custom pipeline available upon request (Variant Explorer RNAseq 2.0). Transcripts were considered differentially expressed when the adjusted *p* values were below 0.05, fold changes exceeded ±1.5, and the false discovery rate (FDR) was below 0.05. For pathway analyses, we tested 10,715 biological pathways from KEGG and GO annotations. Finally, the results were filtered based on an adjusted *p* value below 0.001.

### 2.7. Real-Time PCR

A total of 2 μg of RNA per sample was used for cDNA synthesis following the manufacturer’s protocol, utilizing a high-capacity cDNA reverse transcription kit (Cat #4374967, Thermo Fisher Scientific, Waltham, MA, USA). Significantly differentially expressed genes identified from RNA sequencing were randomly selected, and specific mouse primers (Table 1 (acute) and Table 2 (long-term) for the list) were obtained from the primer bank. These primers were purchased from Oligos, Sigma-Aldrich (Burlington, MA, USA). Quantitative real-time PCR was conducted using an iCycler (Bio-Rad Laboratories, Hercules, CA, USA), with the cycle threshold (Ct) determined by iCycler software version 3. All reactions were performed in triplicate, and Ct values were calculated and normalized to the average Ct of β-actin.

### 2.8. Statistical Analysis

The immunofluorescence and RT-PCR analysis of acute MHV-1 infection data were analyzed using a nonparametric Mann–Whitney test. Similarly, the immunofluorescence and RT-PCR analysis of long-term MHV-1 infection data were analyzed using a nonparametric Kruskal–Wallis test, as performed with GraphPad Prism software version 10.1.2. *p* values less than 0.05 were considered statistically significant. The RNA sequence data were analyzed using the Bioconductor package, which includes DESeq2 (v1.26.0), edgeR (v3.28.1), and limma (v3.42.2). DESeq2 employs a negative binomial distribution with shrinkage estimation, whereas edgeR utilizes empirical Bayes estimation, and limma employs linear models with empirical Bayes moderation via the voom transformation. A correction for testing was applied to adjust the *p*-value and control the false discovery rate(FDR). Pathway analysis uses over-representation analysis or GSEA.

## 3. Results

### 3.1. Immunofluorescence

**Figure 1:**
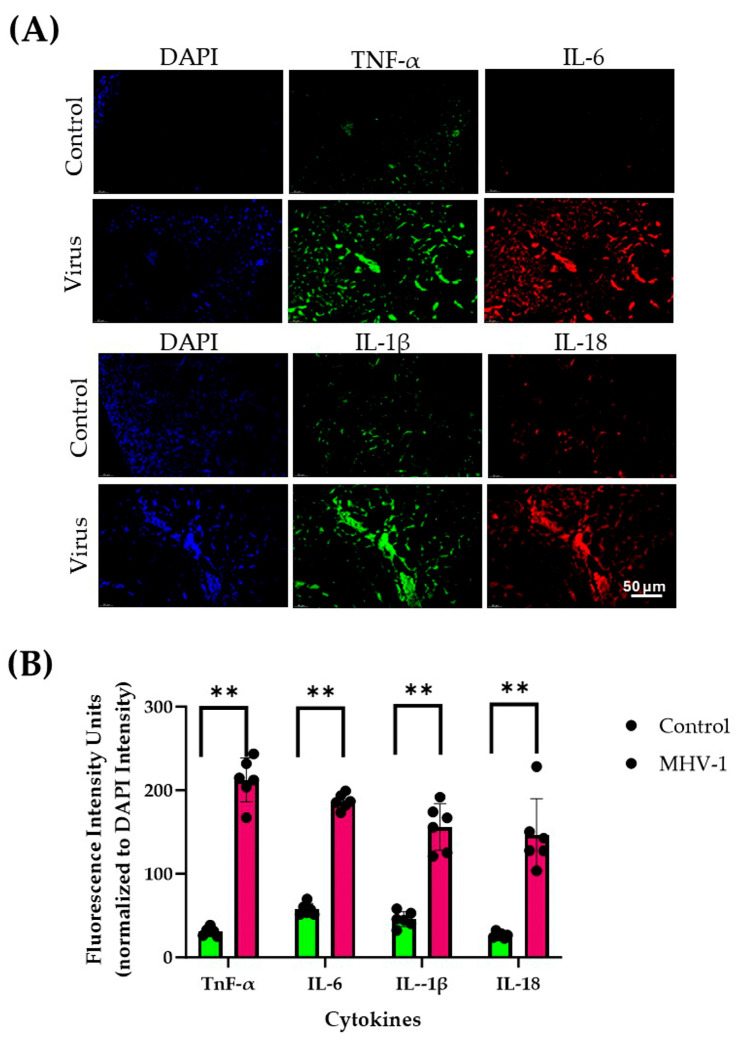
(**A**) Changes in the immunofluorescence levels of IL-1β, IL-6, IL-18, and TNF-α in the kidneys during acute MHV-1 infection (Blue-DAPI; Green—TNF-α, IL-1β (Alexa fluor488); Red-IL-6, IL-18 (Alexa fluor 546). (**B**) Quantitation levels of IL-1β, IL-6, IL-18, and TNF-α are significantly higher in the kidneys of MHV-1-infected subjects compared to controls. Scale bar = 50 μm. The quantitative data were compared using a nonparametric Mann–Whitney test. Error bars represent mean ± SD (*n* = 6). ** *p* ≤ 0.001 indicates a statistically significant difference in MHV-1 compared to controls (Green-Control; Red-MHV-1).

**Figure 2:**
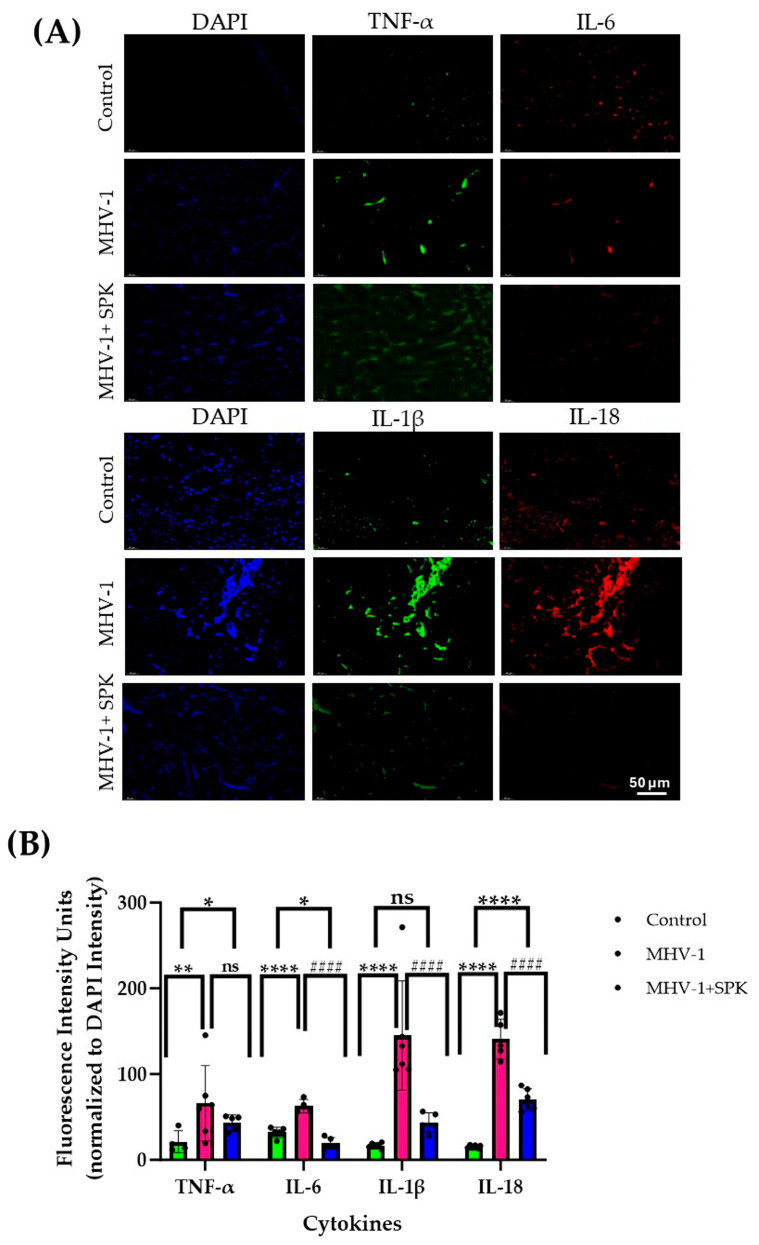
(**A**) Changes in the immunofluorescence levels of IL-1β, IL-6, IL-18, and TNF-α in the kidneys during long-term MHV-1 infection (Blue-DAPI; Green—TNF-α, IL-1β (Alexa fluor488); Red-IL-6, IL-18 (Alexa fluor 546). (**B**) Quantitation levels of IL-1β, IL-6, IL-18, and TNF-α are significantly higher in the long-term kidneys infected with MHV-1 compared to control groups. Scale bars = 50 μm. The quantitative data were compared using a nonparametric Kruskal–Wallis test. Error bars represent Mean ± SD (*n* = 6). * *p* ≤ 0.05; ** *p* ≤ 0.001; **** *p* ≤ 0.0001; ^####^ *p* ≤ 0.0001 are statistically significant in MHV-1 compared to control; ns- non-significant. (Green-Control; Red-MHV-1; Blue-MHV-1+SPK).

**Figure 3:**
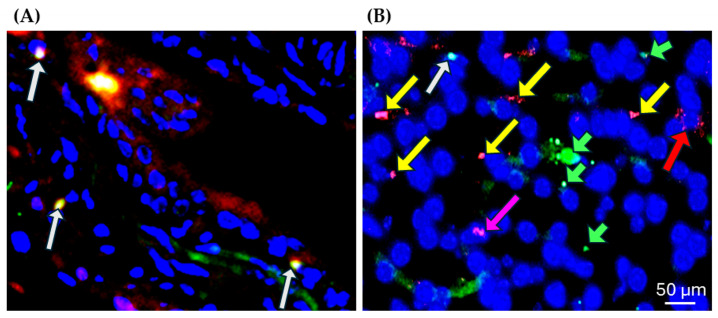
Identification of virus and virus particles in the MHV-1-infected long-COVID kidney using immunofluorescence. (**A**) The white arrow indicates the presence of the virus (spike and nucleocapsid protein) in the kidney of long-COVID MHV-1 infection. (**B**) In addition to the virus (white arrow), we found viral particles (nucleocapsid protein, red) inside the cell (pink arrow) and some on the nuclear membrane (yellow arrow). Some of the nucleocapsid proteins are under degradation (red arrow). Interestingly, we found the S1 protein (green arrow) in the nucleus of the renal cells. Scale bar = 50 µm. Blue (DAPI, 4, 6-diamidino-2-phenylindole); green (spike (S1) glycoprotein); red (nucleocapsid (NC) protein); yellow (colocalization of nucleocapsid protein and spike glycoprotein inside the nucleus and on the nuclear membrane).

Immunofluorescence analysis reveals significantly elevated levels of cytokines, including IL-1β, IL-6, IL-18, and TNF-α, in both acute (Figure 1A,B) and long-term (Figure 2A,B) MHV-1-infected kidneys compared to controls. In addition to cytokine levels, we found the presence of the virus (merged spike and nucleocapsid protein) (Figure 3A) and viral particles (Figure 3B) (spike protein—green; nucleocapsid protein—red) in the renal cells of long-term MHV-1 infection. The continued presence of the virus and viral particles induces an immune response, which is responsible for the persistent inflammation in the kidney. However, the SPK-treated group shows a reduced level of cytokines compared to acute and long-term MHV-1 infection.

### 3.2. mRNA Sequencing

#### 3.2.1. Acute Infection

**Figure 4:**
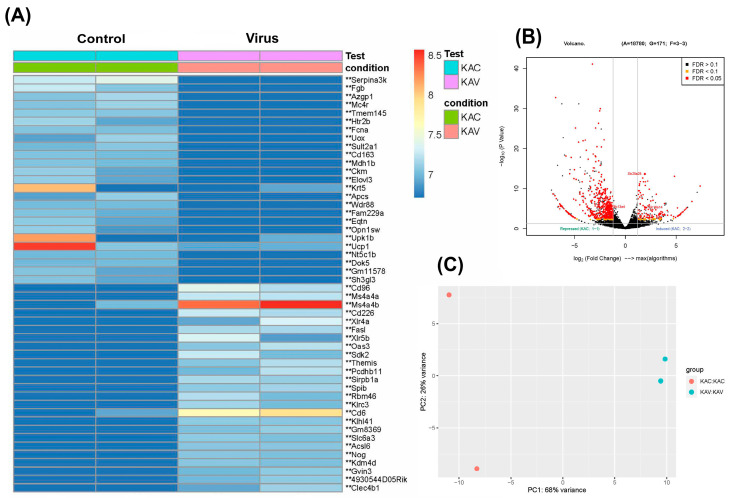
Gene expression profile of acute kidney infection, compared to the control group. (**A**) Heatmap of the genes with the top 50 highest variation in the dataset across all biological replicates. (**B**) The volcano plot shows the distribution of genes between the acute virus and the control group, log_2_ fold change vs. means of normalized counts (−log_10_; *p* value) for DEGs (** *p* ≤ 0.05). (**C**) PCA analysis includes all biological replicates in the acute kidney infection. PC1 accounts for 68% of the variance, and PC2 accounts for 26% of the variance. Acute control vs. virus (*n* = 2).

The heatmap delineates the expression profiles of fifty selected genes within renal tissues from acutely infected murine models. The acute infection is characterized by the upregulation of genes such as *Ms4a4a*, *Ms4a4b*, *Cd6*, *Cd96*, *Cd226*, *Fasl*, *Xlr5b*, *Oas3*, Themis, *Gvin3*, etc. Conversely, genes such as *Ucp1*, *Krt5*, *Upk1b*, *serpina3k*, and *Fgb*, etc. were found to be downregulated in the acute virus group compared to the control (Figure 4A). The volcano plot elucidates the distribution of upregulated and downregulated genes (Figure 4B), while PCA analysis reveals variance between PC1 (68%) and PC2 (26%) (Figure 4C). The pathway analysis explains the GO (Figure S1A) and KEGG (Figure S1B) terms associated with differentially expressed genes in the acute infection.

#### 3.2.2. Long-COVID Infection

**Figure 5:**
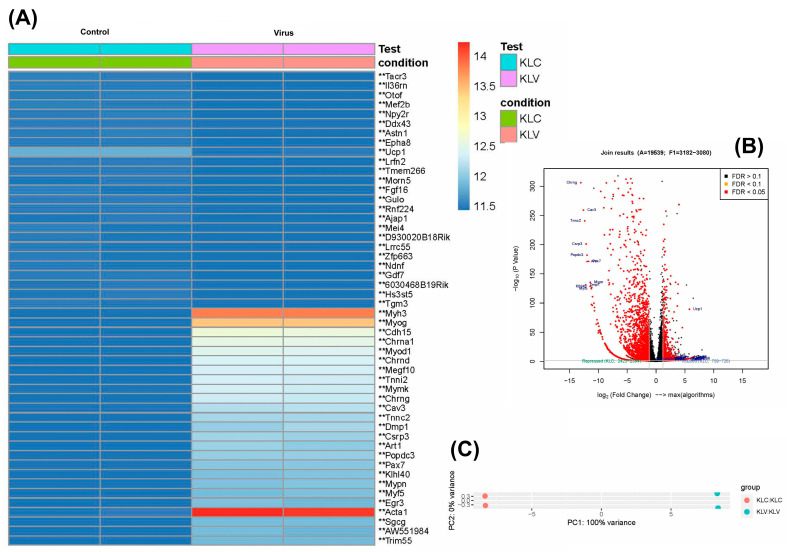
Gene expression profile of long-COVID kidney infection compared to the control group. (**A**) The heatmap displays the top 50 genes with the highest variation across all biological replicates in the dataset (** *p* ≤ 0.05). (**B**) A volcano plot displays the visualized relationship between log_2_ fold change and the normalized counts (−log_10_; *p* value) for differential expression analysis results. (**C**) PCA analysis includes all biological replicates in the long COVID kidney infection. PC1 accounts for 100% variance, and PC2 accounts for 0% variance. Long-term control vs. virus (n = 2).

The heatmap illustrates the expression profile in the kidney tissues of long-term MHV-1 infection compared to the control. In the virus group, several genes are strongly upregulated compared to the control group, suggesting persistent transcriptional alterations long after viral clearance. Notably, *Myh3*, *Myog*, and *Acta1* are significantly upregulated within the virus group. Furthermore, genes such as *chrna1*, *chrnd*, *Mymk*, *Chrng*, *Cav3*, *Tnnc2*, *Dmp1*, *Art1*, *Popdc3*, *Pax7*, *Mypn*, *Myf5*, *Sgcg*, *Cdh15*, *Csrp3*, *Klhl40*, and *Egr3* are upregulated in the virus group (Figure 5A). The volcano plot illustrates the distribution of genes based on log fold change and *p*-value (Figure 5B). Table 3 and Table 4 present the various *SLC* genes that are upregulated and downregulated in the kidneys of MHV-1-infected mice along with their associated pathologies. Furthermore, PCA analysis shows variance between PC1 (100%) and PC2 (0%) (Figure 5C). Pathway analysis delineates the GO (Figure S2A) and KEGG (Figure S2B) terms associated with differentially expressed genes.

#### 3.2.3. Long-COVID with SPIKENET

**Figure 6:**
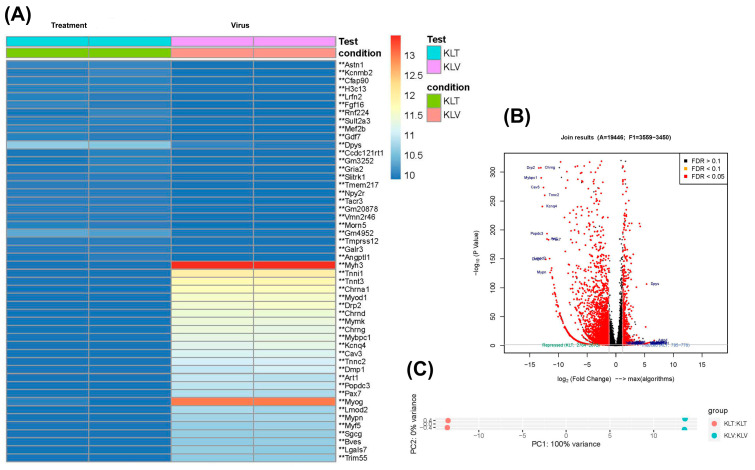
Gene expression profile of long COVID kidney infection, compared to the treatment group. (**A**) The heatmap shows the highest significant gene dataset between viruses and treatment groups (** *p* ≤ 0.05). (**B**) The volcano plot shows the visualized relationship of DEGs between the virus and treatment. (**C**) PCA analysis includes all biological replicates in the long COVID kidney infection and treatment. PC1 accounts for 100% of the variance, and PC2 accounts for 0% of the variance. Long-term treatment vs. virus (*n* = 2).

The heatmap presents the top 50 differentially expressed genes in the virus (long-COVID infection) compared to the treatment (SPK) group. The variances in gene expression highlight that the kidney’s transcriptional landscape responds to persistent viral presence versus therapeutic intervention. The virus group, representing mice with long-term MHV-1 infection, exhibits marked upregulation of several genes, including *Myh3*, *Myog*, *chrna1*, *chrnd*, *Mymk*, *Chrng*, *Cav3*, *Tnnc2*, *Dmp1*, *Art1*, *Popdc3*, *Pax7*, *Mypn*, *Myf5*, and *Sgcg*. Concurrently, these genes are downregulated in the treatment group, comparable to the control (Figure 6A). The volcano plot serves as a representation for the upregulated and downregulated genes based on log fold change and *p* value (Figure 6B), while the PCA analysis elucidates variance between PC1 and PC2 (Figure 6C). Pathway analysis elucidates the GO (Figure S3A) and KEGG (Figure S3B) terms linked to differentially expressed genes.

### 3.3. Altered Expressions of SLC Genes

**Figure 7:**
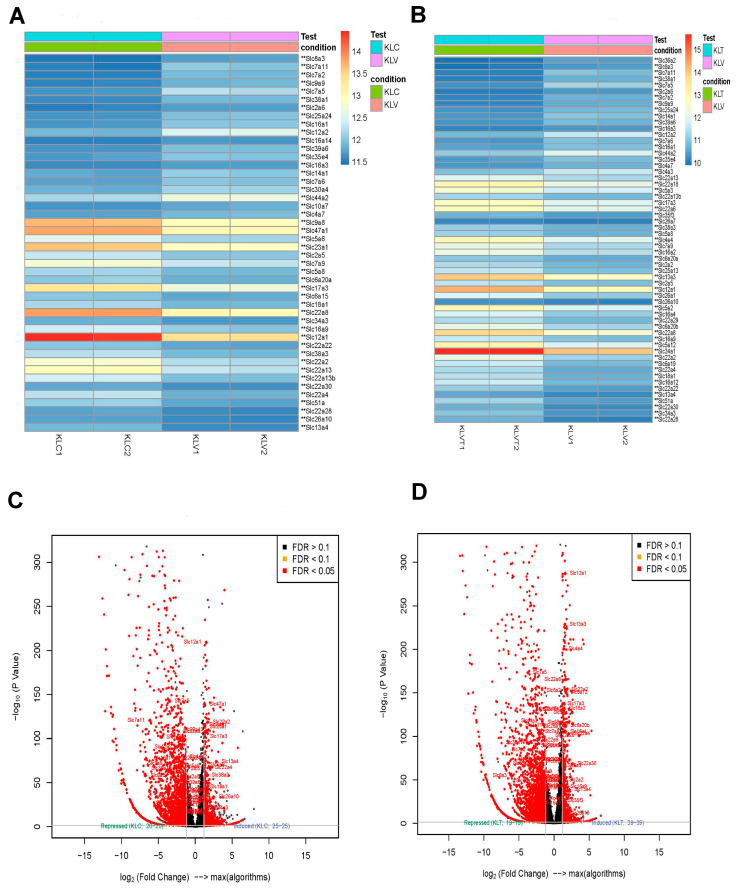
Gene expression profile of SLC genes in the long COVID kidney infection compared to the control and long COVID kidney infection compared to treatment (** *p* ≤ 0.05). (**A**) The heatmap shows the top 50 differentially expressed SLC genes in the virus group compared to the control (*n* = 2). (**B**) Heatmap of the SLC genes shows the top 50 differentially expressed genes in the virus compared to the treatment group (*n* = 2). (**C**) A volcano plot displays the visualized relationship between log_2_ fold change and the normalized counts (−log_10_; *p* value) for differential expression analysis results. (**D**) A volcano plot displays the visualized relationship between log_2_ fold change and the normalized counts (−log_10_; *p* value) for differential expression analysis results.

In addition, we identified differential expressions of 171 solute carrier (SLC) genes in acute MHV-1 infection, in comparison to the control group. Similarly, the long-term MHV-1 renal infection exhibits the differential expression of 65 SLC genes in the virus group compared to the control group (Figure 7A). However, the SLC gene changes were reversed to normal in the treatment group compared to the virus group (Figure 7B). The volcano plot depicts the distribution of upregulated and downregulated genes in both the virus vs. control group (Figure 7C) and the treatment vs. virus group (Figure 7D).

### 3.4. qRT-PCR

**Figure 8:**
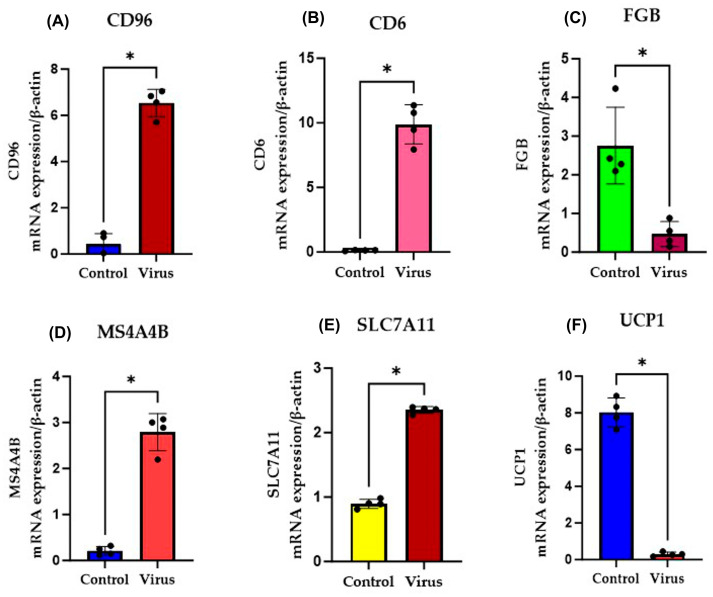
qRT-PCR validation of representative mRNAs from kidney samples acutely infected with MHV-1 compared to control samples. The expressions of (**A**,**B**,**D**,**E**) *CD96*, *CD6*, *MS4A4B*, and *SLC7A11* increased, while (**C**,**F**) *FGB* and *UCP1* decreased at the mRNA level. The quantitative data were analyzed using a nonparametric Mann–Whitney test. Error bars represent mean ± SD (*n* = 4) and * *p* ≤ 0.05 indicates statistical significance (black dots (left)– control group; black dots (right)- virus group; each dot represents the relative expression level).

**Figure 9:**
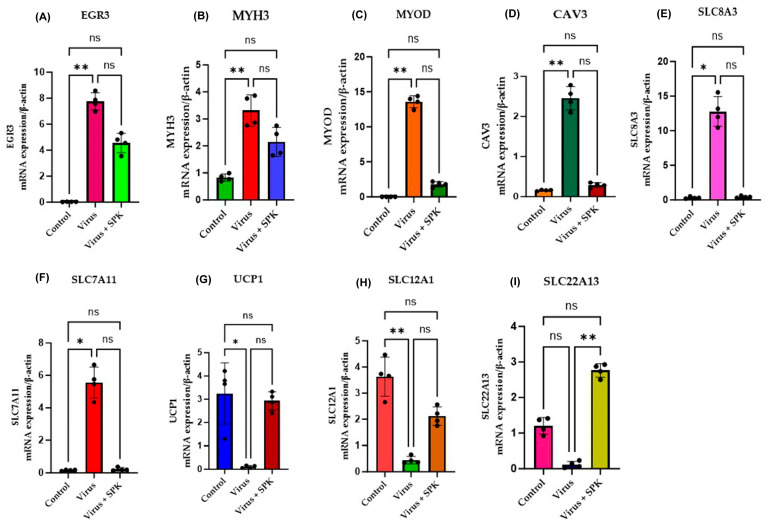
qRT-PCR validation of representative mRNAs from kidney samples infected with MHV-1 after one year compared to control samples. The expressions of (**A**–**F**) EGR3, MYH3, MYOD, *CAV3*, *SLC8A3*, and *SLC7A11* increased, while (**G**–**I**) *UCP1* and *SLC12A1* decreased at the mRNA level. However, SPK normalized the mRNA levels, including *SLC22A13*, to match those of the control. The quantitative data were analyzed using a nonparametric Kruskal–Wallis test. Error bars represent Mean ± SD (*n* = 4) and * *p* ≤ 0.05; ** *p* ≤ 0.001 is statistically significant; ns—nonsignificant (black dots (left)—control group; black dots (middle)—virus group; black dots (right)—virus and SPK group; each dot represents the relative expression level).

The RNA sequence results were further verified using quantitative real-time PCR. The acute kidney shows the upregulation of CD6, CD96, SLC7A11, and MS4A4B (Figure 8) and downregulation of *UCP1* and *FGB* (Figure 8). Similarly, the chronic kidney shows the upregulation of *MYH3*, *MYOD*, *EGR3*, *CAV3*, *SLC8A3*, and *SLC7A11* (Figure 9) and downregulation of *UCP1* and *SLC12A1* (Figure 9) at the mRNA level compared to the control. Thus, the RT-qPCR results revealed that the relative expression of the selected genes was consistent with the RNA sequence data, which demonstrates the reliability of the RNA sequence results.

## 4. Discussion

Our results show the presence of viruses and viral particles in the kidneys of MHV-1-infected mice (7 days and 12 months post-infection). The persistence of MHV-1 leads to immune system dysregulation, resulting in the release of pro-inflammatory cytokines. Furthermore, mRNA sequencing analysis reveals the upregulation of genes involved in inflammation and tissue remodeling in both acute and long-term post-MHV-1 infection. Additionally, we identified changes in numerous SLC genes in these kidney samples, which further affected the reabsorption of soluble proteins. Our findings strongly suggest an inflammatory and immunological response during the acute phase and the long-term post-infection, leading to fibrosis that may contribute to kidney dysfunction in COVID-19.

### 4.1. Cytokine Levels, Virus, and Viral Particles

Cytokine levels are a crucial factor in the renal pathology of COVID-19 patients, compared to other viral infections, resulting in elevated levels of cytokines such as IL-1β, IL-6, IL-18, and TNF-α [15]. Similarly, we found significantly increased expression of cytokines, including IL-1β, IL-6, IL-18, and TNF-α, in both acute and long-term MHV-1-infected kidneys. In our previous report, we found a significantly increased IL-18 mRNA level in long COVID patients with MHV-1 infection, but not in those with acute MHV-1 infection. The difference in protein and mRNA levels of IL-18 is attributed to the instability, translational modifications, and shorter shelf life of mRNA compared to protein [10]. Furthermore, immunofluorescence analysis of kidney tissue reveals the presence of virus and viral particles, S1, and nucleocapsid in the kidney tissue sections of long-term MHV-1-infected animals. This confirmed the continued presence of the virus and its particles, which stimulate an immune response resulting in the release of pro-inflammatory cytokines and persistent inflammation, as well as gene alterations in the kidney. We previously reported the presence of viruses and viral particles in the brains of infants born to mothers infected with SARS-CoV-2 [16]. Additionally, we also found the presence of viral particles in the lung, brain, liver, heart, kidney (acute) [8], and skin (both acute and long-COVID) MHV-1 mouse groups [17]. Furthermore, we now show the presence of both the MHV-1 virus and viral particles in kidney tissues long after infection, and such viral presence and inflammation were absent in SPK-treated mice.

### 4.2. Gene Alterations in Acute and Long-Term Kidney Injury

The acutely infected kidney shows the upregulation of genes involved in immune activation: the *MS4A4A* and *MS4A4B* (*MS4A* gene family), which is linked to leukocyte signaling, while *CD6*, *CD96*, and *CD226* (*DNAM-1*) is a co-stimulatory molecule expressed on T and NK cells, suggesting immune cell recruitment and activation in the kidney during acute infection [18,19,20]. These patterns reflect an increased inflammatory and immune state in response to the viral challenge. Conversely, several genes show downregulation in the infected group relative to controls, indicating possible suppression of normal renal function or immune-regulatory pathways. Genes such as *FGB* (fibrinogen beta chain), *SERPINA3K* (a serine protease inhibitor), and *AZGP1* (a zinc-binding glycoprotein) are downregulated during the acute phase response and potential tissue remodeling processes in the infected kidneys [21,22,23]. In addition, *UCP1*, *UPK1B*, and *CKM* genes, which may be related to metabolic changes, epithelial integrity, or mitochondrial function, are disrupted in MHV-1 infection [24,25,26]. The upregulated expressions of *CD6*, *CD96*, *MS4A4B*, and the downregulation of *UCP1* were further confirmed by RT-PCR.

In the long-term virus kidney group (post 12-month MHV-1 infection), *MYH3*, *MYOG*, and *ACTA1*, involved in muscle development, repair, and cytoskeletal organization, are significantly upregulated [27,28,29]. *ACTA1*, typically associated with skeletal muscle actin, and *SGCG* (Sarcoglycan Gamma), a component of the dystrophin–glycoprotein complex, are often linked to muscle integrity and may indicate cytoskeletal or structural alterations in the kidney post-infection [30]. Additionally, genes linked to muscle structure, regeneration, and contractility, such as *TNNT1*, *TNNT3*, *MYOD1*, *CHRND*, *CHRNA1*, and *MYPN*, are upregulated in the MHV-1-infected long COVID kidney. Also upregulated are *PAX7*, *MYOG*, and *MYF5*, all classic markers of myogenic differentiation and regeneration, indicating either inappropriate activation of muscle-lineage genes or potential cellular stress responses induced by prolonged infection [30,31,32,33,34,35,36]. The high expression of *ANGPT1* and *TMPRSS12* suggests endothelial remodeling and ongoing viral processing activity, respectively, both of which are consistent with chronic viral-induced tissue stress [37]. The mRNA expression profile of *MYOG*, *MYH*, and *MYOD* further confirms the virus-mediated gene alterations in the long COVID kidney mouse model. In contrast, a large cluster of genes, including *TACR3*, *IL36RN*, *MeEF2B*, and *DDX43*, shows consistently lower expression in the long-term infected group compared to controls [33,34,35,36,37]. These genes span functions in neural signaling, transcription regulation, and immune modulation [38,39,40,41,42]. Their downregulation may reflect suppression of normal physiological signaling and homeostatic pathways in chronically affected kidneys. The upregulated genes in the virus group suggest ongoing tissue repair, maladaptive regeneration, or fibrosis, which may contribute to long-term renal pathology if left untreated.

In contrast, the treatment group demonstrates a uniform downregulation of these genes, returning expression levels closer to baseline (healthy control) both at the gene and mRNA level. This normalization across multiple pathways implies that treatment effectively suppressed the abnormal activation of stress-related and regenerative programs. The decreased expression of developmental and cytoskeletal genes in treated samples suggests a significant mitigation of chronic inflammation and damage.

Our findings showcase persistent alterations in renal gene expression following viral infection, indicating lasting effects on kidney health. Furthermore, our results suggest that chronic viral infection can induce a fibrotic renal phenotype, potentially contributing to long-term dysfunction, a key concern for COVID-19 survivors [8,9,10,11]. Gene expression patterns in the infected mice resemble those in chronic kidney disease, supporting the hypothesis that COVID-19 may predispose individuals to renal fibrosis. Early therapeutic intervention appears protective, emphasizing the need for timely treatment. Future research should explore the underlying mechanisms driving viral-induced fibrosis and identify targeted strategies to prevent chronic kidney disease.

### 4.3. Changes in Solute Carrier (SLC) Genes

The kidney serves as a highly versatile organ, with the excretion of any substance by this organ being contingent upon its filtration efficacy, which encompasses reabsorption within the renal tubule and secretion into the tubule lumen. The renal tubule is systematically partitioned into 14 distinct segments, each characterized by the presence of various transporters, including ATPases, ion channels, and solute carriers, which facilitate transport processes [43]. The solute carrier (SLC) family comprises 380 proteins that are responsible for the transport of ions, organic molecules, and the execution of housekeeping functions in the transport of metabolic substrates [43]. Prior investigations have established a robust correlation between the vulnerability of numerous SLC gene loci and the occurrence of metabolic diseases, as well as chronic kidney disease.

Our investigation revealed a considerable differential expression of several solute carrier family genes in the kidneys of both acute and chronically infected mice. In particular, the MHV-1-inoculated mice exhibited an upregulation of the gene families *SLC-2*, *-4*, *-7*, *-8*, *-10*, *-14*, *-16*, *-25*, *-30*, *-35*, and *-38* (Table 3). Conversely, *SLC-22*, *-6*, and *-3*, etc, genes were downregulated in the MHV-1 group (Table 4). Notably, the differentially expressed genes were restored to baseline levels in the SPK-treated group, similar to the control.

**Table 3:** SLC genes upregulated in MHV-1 inoculated mice.

SLC Gene	Subtype	Description	Associated Diseases	References
*SLC2*	*SLC2A6/GLUT6*	Glucose Transporters	Metabolic shift in macrophages	[44]
*SLC4*	*SLC4A7/NBCn1*	Sodium Bicarbonate Cotransporter	Phagosome acidification, pathogen clearance	[45]
*SLC7*	*SLC7A5/LAT1* *SLC7A6/y+LAT1* *SLC7A11/xCT*	Amino acid Transporter	Renal damage /CKD/ Ferroptosis	[46,47]
*SLC8*	*SLC8A3/NCX3*	Na^+^/Ca^2+^ Exchanger	Fibrosis	[48]
*SLC9*	*SLC9A9*	Na^+^/H^+^ Exchanger	Disturbance in pH homeostasis	[49]
*SLC10*	*SLC10A7*	Orphan solute carrier	Reduced calcium influx	[50]
*SLC16*	*SLC16A1/MCT1* *SLC16A3/MCT4* *SLC16A14/MCT14*	Monocarboxylate transporter	Renal cancer	[51]
*SLC25*	*SLC25A24*	Mitochondrial solute transporter	Acute kidney injury	[52]
*SLC38*	*SLC38A1/SNAT1*	Neutral amino acid transporter	Ferroptosis-mediated CKD	[53]
*SLC39*	*SLC39A6/ZIP6*	Zinc transporter	Zinc accumulation	[54]

**Table 4:** SLC genes downregulated in MHV-1 inoculated mice.

Gene	Subtype	Description	Pathology	References
*SLC2*	*SLC2A5/GLUT5*	Fructose Transporters	Fructose-induced hypertension	[55]
*SLC5*	*SLC5A6/SMVT* *SLC5A8/SMCT1*	Vitamin Transporter Lactate and monocarboxylate transporters	Kidney injury Loss of lactate Reabsorption	[56,57]
*SLC12*	*SLC12A1/NKCC2*	Na^+^-K^+^-Cl^−^ cotransporter	Hypotension	[58]
*SLC17*	*SLC17A3/NPT4*	Urate transporter	Hyperurecemia	[59]
*SLC22*	*SLC22A2*, *SLC22A4*, *SLC22A8/OAT3*, *SLC22A13/OAT10*, *SLC22A13B*, *SLC22A22*, *SLC22A28*	Cation/anion transporter	Elevated blood pressure and CKD	[60,61,62,63]
*SLC23*	*SLC23A1/SVCT1*	Vitamin C transporter	Renal leak of vitamin C, low hemoglobin	[64]
*SLC34*	*SLC34A3/NPT2c*	Sodium/Phosphate Cotransporter	Hypophosphatemia	[65]
*SLC38*	*SLC38A3/SNAT3*	Neutral amino acid (Glutamine) transporter	Deficiency in renal ammonia and urea excretion	[66,67]
*SLC47*	*SLC47A1/MATE1*	Multidrug/toxin extrusion protein 1	Drug toxicity	[68]
*SLC51*	*SLC51A/OST* *α*	Organic solute transporter α	Bile acid accumulation in the kidney	[69]

The *SLC8* gene family encodes the sodium calcium exchanger *(NCX*), which is responsible for facilitating the transport of Ca^2+^ across the cellular membrane. Three distinct SLC genes encode: *SLC8A1 (NCX1)*, *SLC8A2 (NCX2)*, and *SLC8A3 (NCX3)*. The NCX is critical for calcium reabsorption within the kidney [48]. Our findings indicate an increased expression of *SLC8A3* in the kidneys of long-term MVH-1-affected mice, which was further confirmed by RT-PCR. Conversely, a significantly reduced expression of *SLC8A1* was noted during acute MHV-1 infection. Additional research is warranted to elucidate the upregulation and functional significance of SLC8A3 in both murine and human models. The expression of *SLC4A7 (NBCn1)* was found to be elevated within the viral group. NBCn1 represents an acid–base transporter that facilitates the entry of Na^+^ and HCO_3_^−^ into the cell in response to fluctuations in pH or during NaCl or NaHCO_3_ overload, thereby regulating renal acid–base equilibrium [45,70]. Dysfunction of SLC4A7 is implicated in renal sodium retention and the subsequent development of hypertension [58,71]. Furthermore, we observed an upregulation of *SLC10A7* within the viral cohort. *SLC10A7* is classified as an orphan member of the *SLC10* family of bile acid transporters, and its overexpression leads to a reduction in calcium influx, consequently lowering intracellular calcium concentrations [50]. In a similar vein, we identified an upregulation of the SLC5 gene family member, *SLC5A12*, within the long COVID treatment group, in contrast to the downregulation of *SLC5A12* and *SLC5A8* observed in the viral group.

The *SLC7* family represents a substantial cohort of secondary active transport proteins that facilitate uniport, symport, and antiport mechanisms for various cellular proteins across cellular membranes. This family is categorically subdivided into two distinct groups: L-type amino acid transporters (LAT) and cationic amino acid transporters (CAT) [47,72]. Our findings indicate an upregulation of *SLC7A2 (CAT2)*, *SLC7A5 (LAT1)*, *SLC7A6 (y+LAT2)*, and *SLC7A11* (xCT), alongside a downregulation of *SLC7A9* (b+AT) in mice inoculated with MHV-1. This transport mechanism is integral for facilitating cystine transport, which is necessary for the biosynthesis of GSH and glutamate. The xCT protein is identified as being upregulated in cells exhibiting a heightened requirement for GSH. The *SLC7A9* gene plays a crucial role in the transport of cationic amino acids and L-cystine, in conjunction with the auxiliary protein *SLC3A1*. A reduction in *SLC7A9* expression has been associated with the pathogenesis of urinary cystine calculi [47].

Our investigation also revealed the downregulation of multiple genes within the *SLC22* family, specifically *SLC22A2 (OCT2)*, *SLC22A4 (OCTN1)*, *SLC22A8 (OAT3)*, *SLC22A13 (OAT10)*, *SLC22A13b*, *SLC22A22 (oatPG)*, *SLC22A28*, and *SLC22A30*. The *SLC22* family comprises multi-specific transporters that are pivotal in the translocation of small endogenous metabolites, pharmaceuticals, and toxins, encompassing organic anions (OAs) and organic cations (OCs). Prior studies have established that the downregulation of *SLC22* genes serves as a prevalent indicator of acute renal injury and subsequent recovery. Urinary fragments of OATs are recognized as biomarkers for acute kidney injury [60,61,62,63]. *SLC22A2* functions as the primary organic cation transporter (OCT) that is predominantly localized in the basolateral membrane of proximal tubular cells, thereby playing a significant role in regulating renal uptake and nephrotoxicity [49]. The downregulation of *OCT2* has been documented in both rat and mouse models of chronic kidney disease (CKD) or acute kidney injury (AKI), as well as in human patients with CKD, primarily attributed to inflammatory processes and oxidative stress [50]. The SLC downregulation results in the elevation of blood pressure, which shows the link between renal damage and cardiovascular system dysfunction. Additionally, we observed a downregulation of *SLC12A1/NKCC2* within the viral group, which was further confirmed by RT-PCR. *NKCC2* serves as a critical regulator of sodium reabsorption and blood pressure. The downregulation of *NKCC2* is associated with the onset of polyuria and hydronephrosis [58,73].

Furthermore, our analysis identified a downregulation of *SLC34A3* in the MHV-1-infected group. The *SLC34A3* gene encodes a sodium-phosphate cotransporter protein that facilitates the movement of phosphate into renal tubular cells, helping to maintain intracellular osmolality. The decrease in *SLC34A3* results in increased phosphate excretion in urine and disrupts phosphate homeostasis [65,74]. Conversely, *SLC34A3* was found to be upregulated in the SPK treatment group, which is linked to the restoration of phosphate balance.

The *SLC38* family comprises eleven members that are involved in transporting glutamine and neutral amino acids, which are essential for cell growth, invasion, migration, and the formation of new blood vessels. *SLC38A1* has a high affinity for glutamine, enabling it to transport in one direction. Its increased expression is linked to heightened cell metabolism during cancer progression [75]. However, its specific roles in the kidney are not yet fully understood. We found that *SLC38A3/SNAT3* decreased in the viral group. *SNAT3* mainly transports various amino acids, including glutamine, which is vital for cell and organ function. The reduction in *SNAT3* leads to changes in urea and ammonia excretion [66,67]. Finally, ongoing viral presence, persistent inflammation, and alterations in *SLC* gene expression in the kidney may lead to chronic kidney disease and hypertension (cardiovascular problems), which can further impact the autonomic nervous system. This highlights the connection between the kidney, heart, and brain (kidney–heart–brain axis) [76,77,78].

### 4.4. Strength of the MHV-1 Model

The MHV-1 model is considered a suitable mouse model for studying both acute and long COVID infections. To the best of our knowledge, there are more than 30 articles that demonstrate a significant correlation between this model and humans infected with SARS-CoV-2, and over 50 articles that have reported the likelihood of this model replacing many of the existing ones for COVID-19 studies. This model has been well-characterized for (1) immune response; (2) multiorgan pathology, including lung, heart, kidney, and intestine; (3) clinical symptoms; and (4) animal survival associated with COVID-19 in humans [5,6,7,8,9,10,11,12]. Regarding the reliability of chronic conditions, although researchers are skeptical about using MHV-1 to study human COVID-19 due to the difference in receptors (ACE2 vs. CEACAM1), the severity, clinical, pathological, immunological, and functional features observed in MHV-1 more closely recapitulate changes seen in humans with SARS-CoV-2. These include the K18-hACE2 transgenic mouse model, the mouse-adapted SARS-CoV-2 model, the endogenous mouse ACE2 promoter model, the adenovirus hACE2 mouse model for SARS-CoV-2 infection, ferret models, and nonhuman primate models [5,6]. The reason these models are considered more suitable than MHV-1 is that the severity of death is more significant compared to humans hospitalized with severe SARS-CoV-2 infection. While the Syrian hamster model is recommended for studying the mechanisms and consequences of COVID-19, it is not ideal because it lacks histopathological changes in the brain, liver, heart, and kidney. This limits the hamster model for studying extrapulmonary pathologies observed in COVID-19 patients. Other recent models, such as mouse-adapted MA10 SARS-CoV-2 in BALB/c and C57BL/6 mice, are also used to study acute and long-term COVID-19. However, these models have limitations because these mice did not develop lung pathology like humans and did not survive longer than the MHV-1 model. In our earlier studies, we found that MHV-1 infection led to acute and chronic kidney injury, including acute tubular injury, collapsing glomerulopathy, proximal and distal tubular necrosis, vascular congestion, epithelial degeneration, vacuolation, interstitial hemorrhage, and inflammation characterized by macrophage and lymphocyte infiltration [7,8,9,10], consistent with studies in humans with COVID-19, which includes human kidney biopsy samples [79,80,81,82]. Moreover, our unpublished brain gene expression analysis shows a strong correlation between MHV-1 brain and the brains of humans with various neurological conditions associated with COVID-19. These findings strongly support the use of the MHV-1 mouse model for studying COVID-19.

We used female A/J mice in this study. Although infection rates are similar between genders, men face a higher risk of worse outcomes and death regardless of age. This is likely due to differences in immune response, ACE2 receptor expression, hormonal influences, and behavioral factors in COVID-19. We and others have used female mice with higher estrogen levels, which show better results and are relatively resistant to severe disease and death following MHV-1, unlike males. More specifically, the pathological changes we observed in females are closer to those found in humans with COVID-19 [7,8,9,10,11,12] than in males. We now also see better functional outcomes in females compared to males based on human data.

### 4.5. Limitations

This study has certain limitations. We and others have identified many factors relevant to humans with COVID-19 post-MHV-1 infection in A/J mice, both during the acute phase and in the long-term post-infection. Strain-dependent pathology is the key issue. Different inbred mouse strains show varying susceptibilities and disease outcomes with MHV-1. For example, A/J mice have moderate mortality, whereas C57BL/6 mice experience mild illness. This variability complicates the interpretation and generalization of results. Therefore, it would be helpful to evaluate its effects across a broad range of animal species, despite differences in receptor mechanisms and the reasons for such differences, which could offer clues as to why SARS-CoV-2 infection varies among humans. However, it is highly unlikely to conduct any human studies at this stage due to the limited number of hospital admissions with severe COVID-19.

## 5. Conclusions

Overall, the gene expression profile in acute virus mice indicates robust transcriptional activation of immune response genes and suppression of markers associated with normal renal homeostasis. This suggests that acute coronavirus infection triggers a significant inflammatory and immunological response in the kidney, contributing to the observed pathology during acute kidney injury. The long-term MHV-1 infection leads to changes in the gene expression profile of kidney regeneration, ultimately contributing to the development of fibrosis.

Furthermore, we observed the overexpression of numerous SLC genes in both the acute and long-term MHV-1-infected virus groups. The infected kidney exhibits overexpression of SLC genes, including SLC2, SLC4, SLC7, SLC8, SLC10, SLC14, SLC16, SLC25, SLC30, SLC35, and SLC38, in the MHV-1 inoculated mouse group. The upregulated SLC genes result in changes in the transport of sodium, calcium, amino acids, and sugars. At the same time, SLC22, SLC6, and SLC3 genes are downregulated in the MHV-1 group. Downregulation of SLC22 is considered a common indicator of kidney injury and is highly susceptible in patients with CKD. However, the number of differentially expressed genes was reduced to normal levels by the SPK treatment group compared to the virus. This demonstrates the effect of SPK in preventing MHV-1 virus-mediated kidney damage by reducing the elevation of virus-mediated inflammation in the mouse model. However, further study is recommended to measure proteinuria, serum creatinine, and blood urea nitrogen (BUN) and to confirm the SPK role in the treatment of long-COVID infection in humans.

## Figures and Tables

**Table 1:** Primers used in the RT-PCR analysis of acute kidney.

Gene Name	Gene Description	Primer Sequence
*UCP1*	Uncoupling Protein 1	AGGCTTCCAGTACCATTAGGT CTGAGTGAGGCAAAGCTGATTT
*MS4A4B*	membrane-spanning 4-domains, subfamily A, member 4B	TGACACTTCAACCATTGCTACC ACACATTTCCTGGAACATTGGTC
*CD6*	Cluster of Differentiation 6	GGAGGGCTACTGCAATGATCC GTGAGGGGACTCTTCTCAGAAT
*CD96*	Cluster of Differentiation 6	TGGGAAGAGCTATTCAATGTTGG AGAGGCCATATTGGGGATGATAA
*Fgb*	fibrinogen B beta (Bβ) chain	ACGATGAACCGACGGATAGC CCGTAGGACACAACACTCCC
*SLC7a11*	cystine/glutamate antiporter	GGCACCGTCATCGGATCAG CTCCACAGGCAGACCAGAAAA

**Table 2:** Primers used in the RT-PCR analysis of long-COVID kidney.

Gene Name	Gene Description	Primer Sequence
*Myh3*	Myosin-3	AAAAGGCCATCACTGACGC CAGCTCTCTGATCCGTGTCTC
*UCP1*	Uncoupling Protein 1	AGGCTTCCAGTACCATTAGGT CTGAGTGAGGCAAAGCTGATTT
*Myod1*	Myogenic Differentiation 1	CCACTCCGGGACATAGACTTG AAAAGCGCAGGTCTGGTGAG
*Cav3*	Caveolin 3	GGATCTGGAAGCTCGGATCAT TCCGCAATCACGTCTTCAAAAT
*Egr3*	Early Growth Response 3	CCGGTGACCATGAGCAGTTT TAATGGGCTACCGAGTCGCT
*SLC8A3*	sodium/calcium exchanger 3	CCCCCGCATGGTGGATATG CCCTCCTGCACTAACAGTGA
*SLC12A1*	sodium–potassium–chloride cotransporter	TCATTGGCCTGAGCGTAGTTG TTTGTGCAAATAGCCGACATAGA
*SLC22A13*	Organic Anion Transporter	TTCAGGTGCGGTTGACCATC GTGGTTCTTAACCCAGGACAC
*SLC7a11*	cystine/glutamate antiporter	GGCACCGTCATCGGATCAG CTCCACAGGCAGACCAGAAAA

## Data Availability

The data presented in this study are available upon request from the corresponding author. However, due to the University of Miami Miller School of Medicine’s privacy policy, they are not publicly available.

## Supplementary Materials

The following supporting information can be downloaded at: https://www.mdpi.com/article/10.3390/diseases13080246/s1.

## Abbreviations

The following abbreviations are used in this manuscript:

AKI	Acute kidney injury
Azgp1	Zinc-binding glycoprotein
CAT	Cationic amino acid transporters
CKD	Chronic kidney disease
COVID-19	Coronavirus Disease-2019
DEGs	Differentially expressed genes
ESRD	End-stage renal disease
FDR	False discovery rate
FGB	Fibrinogen beta chain
GSH	Glutathione
LAT	L-type amino acid transporters
MCT	Monocarboxylate transporters
MHV-1	Murine hepatitis virus-1
NC	Nucleocapsid
NCX	Sodium-calcium exchanger
NGS	Next-generation sequencing
OAs	Organic anions
OATs	Organic anion transporters
OCs	Organic cations
OCT	Organic cation transporter
QC	Quality control
S1 protein	Spike 1 protein
SARS-CoV-2	Severe acute respiratory syndrome coronavirus 2
Serpina3k	Serine protease inhibitor
SLC	Solute carrier
SPK	Spikenet
TPM	Transcripts per million
