# Supplementary material for: Persistent Inflammation, Maladaptive Remodeling, and Fibrosis in the Kidney Following Long COVID-like MHV-1 Mouse Model
Source: Diseases. 2025 Aug 5;13(8):246. doi: 10.3390/diseases13080246 (PMC12385743; doi:10.3390/diseases13080246)
Supplement: Supplementary file 1 [file diseases-13-00246-s001.zip › diseases-3669959-supplementary.pdf]

Supplementary file:

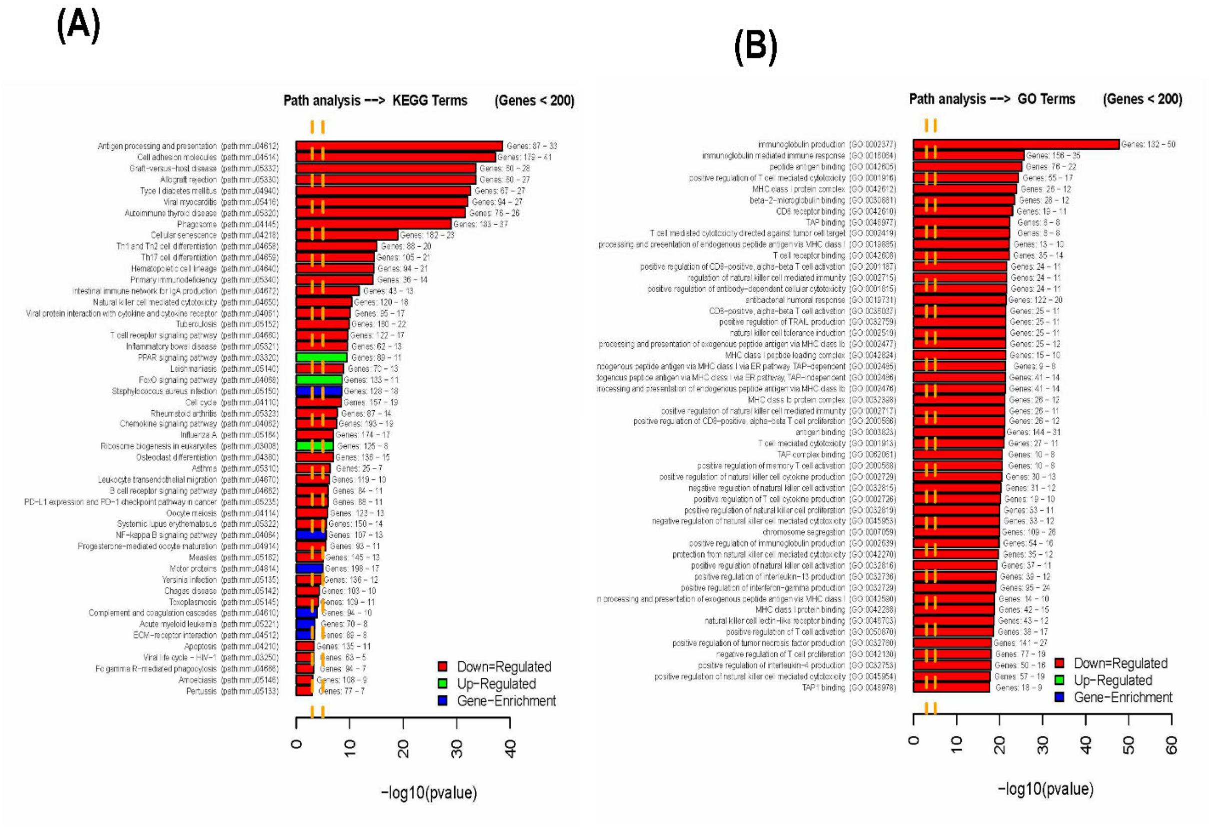

Figure S1: Go and KEGG analysis of acute kidney infection. **(A)** KEGG pathway analysis in the acute kidney infection. The 50 most significantly (P<0.05) enriched KEGG pathways for upregulated (Green), downregulated (Red), and Gene enrichments (Blue) are presented. **(B)** GO enrichment analysis of DEGs in the acute kidney infection. The 50 most significantly (P<0.05) enriched GO terms for upregulated (Green), downregulated (Red), and Gene enrichments (Blue) are presented.

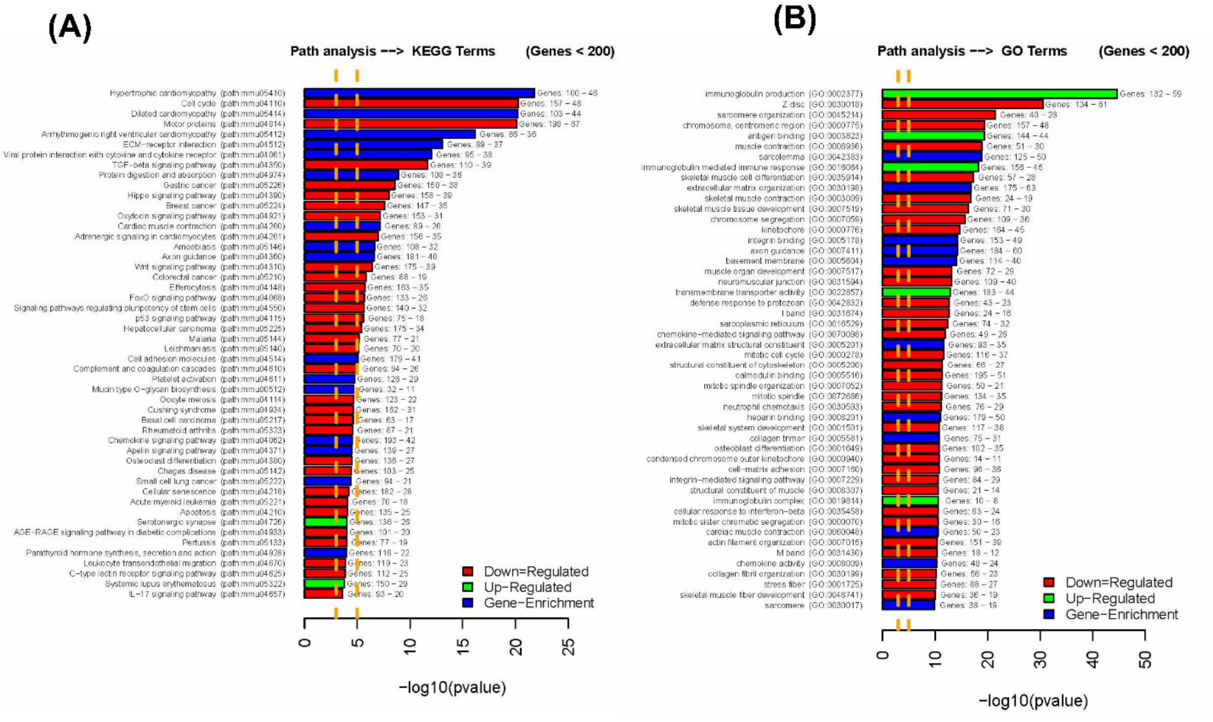

Figure S2: (A) KEGG pathway analysis in Long COVID kidney infection. The 50 most significant (P<0.05) enriched KEGG pathways for upregulated (Green), downregulated (Red), and gene enrichments (Blue) are presented for DEGs. **(B)** GO enrichment analysis of DEGs in the Long COVID kidney infection. The 50 most significantly (P<0.05) enriched GO terms for upregulated (Green), downregulated (Red), and Gene enrichments (Blue) for DEGs.

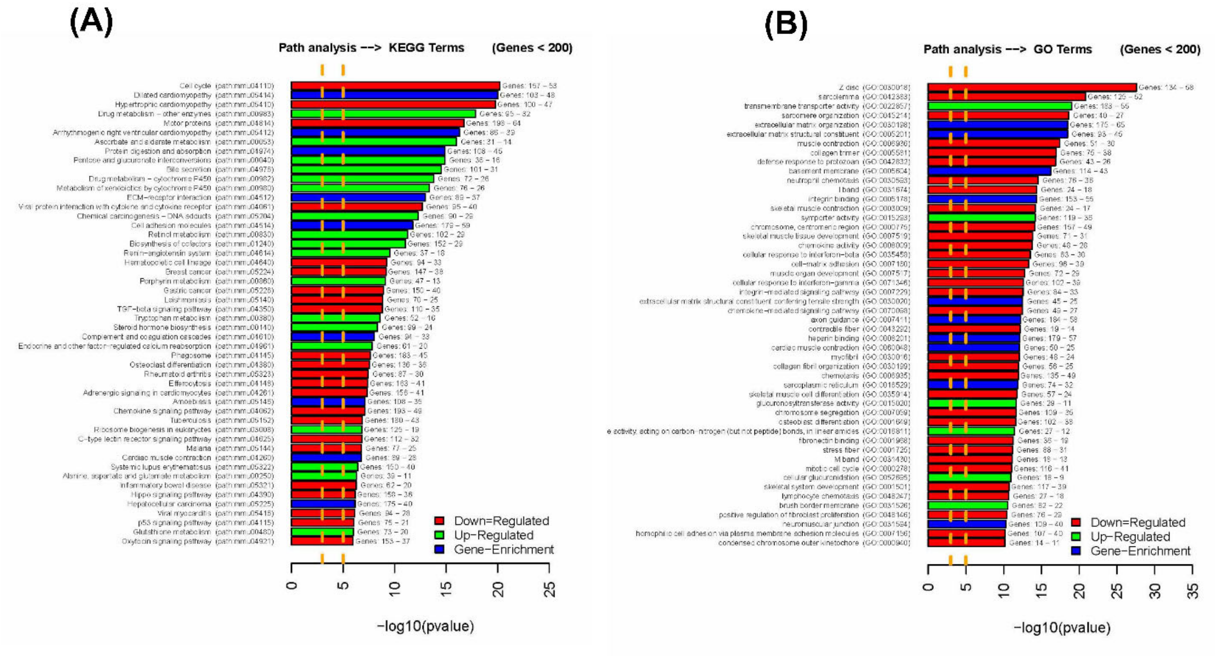

Figure S3: (A) KEGG pathway analysis in Long COVID kidney infection. The 50 most significant (P<0.05) enriched KEGG pathways for upregulated (Green), downregulated (Red), and gene enrichments (Blue) are presented for DEGs. **(B)** GO enrichment analysis of DEGs in the Long COVID kidney infection. The 50 most significantly (P<0.05) enriched GO terms for upregulated (Green), downregulated (Red), and Gene enrichments (Blue) for DEGs.
